# The climatic and river runoff trends in Central Asia: The case of Zhetysu Alatau region, the south-eastern part of Kazakhstan

**DOI:** 10.1016/j.heliyon.2023.e17897

**Published:** 2023-07-05

**Authors:** Sabira Issaldayeva, Sayat Alimkulov, Zhanar Raimbekova, Roza Bekseitova, Marat Karatayev

**Affiliations:** a Al-Farabi Kazakh National University, School of Geography and Environmental Management, Al-Farabi Ave. 70, A15E3C7, Almaty, Kazakhstan; b Institute of Environmental Management, Slovak University of Agriculture, Tr. A. Hlinku 2, 949 76, Nitra, Slovak Republic; c Institute of Geography and Water Security, Water Research Laboratory, Kabanbai Batyr Str., 67, Almaty, A25T7A1, Kazakhstan; d Institute of Environmental Systems Sciences, Karl-Franzens University of Graz, Merangasse 18-1, A-8010, Graz, Austria; e Centre for the Environment, University of Nottingham, University Park, NG7 2RD, Nottingham, UK

**Keywords:** Water resources, Climate parameters, Trend analysis, Zhetysu alatau, Kazakhstan

## Abstract

This study analyses, compares and correlates historical hydrometeorological data for the Zhetysu Alatau region and its main rivers (Lepsy, Sarykan, Koktal, Byzhy) to document characteristics and evidence of changes in climate change (temperature and precipitation) and its impact on annual and monthly river runoff. This study applies Andreyanov method for computation of runoff data and Mann-Kendall statistic method for assessing statistically significant or weak trends. The study reveals that the pattern of temperature for period of 1960–2020 and runoff for period of 1930–2019 has changed in the region. Mann-Kendall test result indicates a statistically significant increase in temperature at all meteorological stations (p<0.01), while the fluctuations in precipitation trends are not meaningful (p>0.05). Andreyanov method shows significant changes in intra-annual runoff trends, e.g., calculations for the period of 1965–2019 show a decrease of 5.3% in summer runoff in the Sarykan river, and the increase in runoff in the remaining months was 6.4% higher compared to the period of 1930–1965. Furthermore, the Mann-Kendall test confirms a significant positive trend in the change of seasonal runoff for the Sarykan, Byzhy, and Koktal rivers (p<0.02). The precipitation is one of the main factors influencing river runoff and the correlation coefficient between river runoff and precipitation for Lepsy river is r=0.81; for Byzhy river is r=0.70; for Koktal river is r=0.62; for Sarykan river is r=0.60.

## 1 Introduction

The environment in all regions of the world is changing quickly due to anthropogenic activities and environmental change [1,2]. The element most impacted by anthropogenic activities and environmental changes is surface water resources [3]. Examples of anthropogenic activities that have changed spatial and temporal distribution of water resources are agriculture, irrigation, land use, water-soil conservation, and the energy sector [4,5]. Environmental change appears as variations in temperature and precipitation patterns, and as a result, it directly affects the hydrological and thermal characteristics of water resources and freshwater ecosystem quality [6,7], while anthropogenic activities accelerate these changes [[8], [9], [10]]. The impact of environmental change on river flow varies depending on the geographic region [11]. In cold areas, runoff and its features are significantly impacted by rising temperatures [12]. Temperature variations affect the timing of snowmelt runoff peaks and the increase of winter and spring runoff [13]. The flooding situation in humid places could get worse due to a rise in the frequency of extreme precipitation events [14]. The regional water scenario worsens in semi-arid regions as temperatures rise and evaporation rates rise [15]. The river flows in arid regions depend on the impact of climate change on glaciers, the primary source of water supply for surface water resources [[16], [17], [18], [19], [20]].

The Central Asian water resources are especially vulnerable because this region primarily depends on snow and glacier replenishment in the mountainous areas [21]. It is reported that several surface water resources of Central Asian region have experienced sharp changes in runoff trends [22]. As a result, surface-water resources surrounding ecological environment on these rivers has been somewhat impacted [23]. Changes in the runoff trends are a significant factor in the sustainable development of Central Asian agriculture [24]. Negative runoff changes influenced regional stability and national security, exacerbated water resource conflicts, and strained international relations in Central Asian region [25,26]. A rapid population increase, and irrational use of local water supplies have severely hampered economic progress and ecological preservation in the Central Asian region [27]. Due to the escalating water shortage concerns and increasing frequency of floods and droughts, the effects of anthropogenic activities and climate variability on surface water resources have emerged as a crucial area of sustainable management policy [28]. The regional climate and water resource changes of Central Asia have been investigated to assure ecological and water security [29], develop regional climate changes for water resource impact assessment, and better comprehend the policies regulating changes to the region's water resources [[30], [31], [32]]. However, researching a hydrologic response to climate change and variation is needed for the Central Asian region for sustainable development and science-based policy formulation and implementation. Therefore, ensuring water security in the face of these challenges requires a monitoring runoff, which is a critical activity for ensuring the sustainable and effective management of water resources.

This study analyses, compares and corelates a historical hydrometeorological data for the Zhetysu Alatau region and its main four rivers (Lepsy, Sarykan, Koktal, Byzhy) for documentation of characteristics and evidence of changes in climate change (temperature and precipitation), and its impact on annual and monthly river runoff. By using a combination of methods (method of reconstruction of missing data, Andreyanov method, Mann-Kendall method), it is possible to gain a more comprehensive understanding of runoff patterns and its dynamics, and to develop effective strategies for addressing water-related challenges. The rest of the study is organized into three sections. Section 2 presents a description, information, and data on the study area, hydrometeorological data collection and reconstruction of missing data, Andreyanov and Mann-Kendall methods. Next, in section 3, the study provides results of data analysis on temperature and precipitation change, temporal runoff dynamics, and comparative description of intra-annual runoff change in Lepsy, Sarykan, Koktal, Byzhy rivers. Finally, section 4, 5 provides discussion, and it draws a conclusion.

## 2 Study area, datasets and methodology

### 2.1 Study area description

**Fig. 1:**
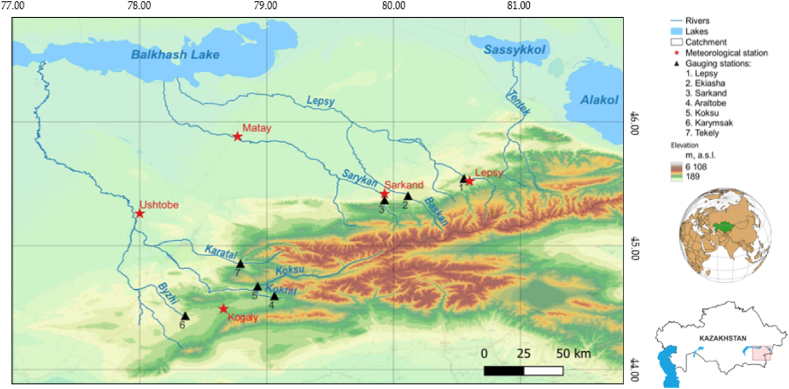
Study area: the Zhetysu Alatau, the South-Eastern part of Kazakhstan.

The Zhetysu Alatau region of Central Asian region (Fig. 1) is the research subject of this paper. The Zhetysu Alatau area is a mountain and river basin located in the South-Eastern part of Kazakhstan between the Ile River and Alakol lake along the state border between Kazakhstan and China. The Zhetysu Alatau Mountains span 450 latitude km and have a width of 100–250 km [33]. The name of Zhetysu Alatau means “Seven Rivers Mountains” in Kazakh, referring to the seven rivers that originate from the range and flow into different directions. All rivers differ regarding observation periods, water catchment areas, and orographic and landscape locations. The Lepsy river, which is situated in glaciers at an elevation of more than 3000 m above sea level, is the most significant in the northeast zone [34]. The Sarykan and Koktal, rivers, which have their origins in glaciers as well, flow in the northern zone. The Byzhy river is situated in northwest slope with dominant snow cover. According to the hydrological regime, most rivers are spring-to-summer seasonal flood streams, with maximum discharges occurring when the snow cover melts in the spring and glaciers feed rivers in the summer. These rivers play an important role in the functioning of the large endorheic lake Balkash Lake. The Balkhash Lake is a main benefiter of the runoff of the rivers of the Zhetysu Alatau. In the context of a reduction in the inflow of water along the Ile River from the territory of China, the ways to preserve Balkash Lake will largely depend on the rivers under study [34].

The continental climate of the Zhetysu Alatau mountain system is characterized by a significant amount of solar radiation, a dry and warm summer, a cold winter with minimal snow in the foothills, frequent temperature inversions, and a heavy coating of snow in the mountains. The coldest month is January, which ranges from −13 °C to −14 °C. The typical annual temperature in the high-altitude region of Zhetysu Alatau is between −5 °C and −7 °C [35]. Both arctic and temperate air masses influence the Zhetysu Alatau mountain system's climate. During the winter, Arctic air masses flow from the north and northwest, decreasing local air temperatures [36]. The altitude impacts the distribution of precipitation. Precipitation ranges from 1000 to 1600 mm^−1^, with the peak amounts occurring between 1800 and 2200 m above sea level [37]. The Zhetysu Alatau range is home to several glaciers [38]. The largest glacier in the Zhetysu Alatau range is the Inylchek glacier, which spans both the Kyrgyzstan and Kazakhstan borders and is part of the greater Tian Shan mountain range. Other glaciers in the Zhetysu Alatau range include the South Inylchek glacier, Kaindy glacier, and the Merzbacher glacier. The Balkhash Lake is a main benefiter of the runoff of the rivers of the Zhetysu Alatau [39]. The area is also rich in biodiversity, with numerous endemic and rare species of plants and animals, including the endangered snow leopard, ibex, and argali sheep [40].

### 2.2 Hydrometeorological data

Hydrological data for the period 1930–1965 was obtained from the reference document “Surface water resources of the USSR” [41]. Data from 1965 to 2020 was provided by the National Hydrometeorological Agency “Kazhydromet”. The data includes hydrological indicators for Zhetysu Alatau rivers. In addition, monthly climate data (temperature and precipitation) for the period 1960–2020 were collected from several meteorological stations in Kogaly, Lepsy, Matay, Sarkand, Ushtobe. For a few stations of the study region, there needs to be more hydrologic data on mean monthly and annual runoff data. For reconstruction runoff data, hydrologic method was used based on the similarities between hydrologic phenomena [[42], [43], [44]]. This method is advised for use by the WMO's and Hydrological Practices and Kazakhstan's national hydrometeorological guidelines [45]. The primary factor in selecting a similar site is the existence of synchronicity in the variations of the river runoff of the calculated reference and analog station, which is quantified by the coefficient of pair or multiple correlations between the runoff at these places using regression analysis. In the regression analysis of monthly runoff data for year ($Q_{i}$) the following model was used in this study:

(1) $$n^{,}\geq \left(6-10\right);R\geq R_{kp};\frac{R}{\sigma_{R}}\geq A_{kp};\frac{k}{\sigma_{k}}\geq B_{kp}$$

where $n^{,}$ - number of years of observations at the reference and analog station ($n^{,}$ - ≥ 6 with one analog, $n^{,}$ > = 10 with two or more analogues); R – coefficient of pair or multiple correlation between the runoff values of the studied river and runoff values at analog points; k – regression coefficient; $\sigma_{k}$ – standard error of the regression coefficient; $R_{kp}$ – critical value of the pair or multiple correlation coefficient; $A_{kp}$, $B_{kp}$ – critical values of $\frac{R}{\sigma_{R}}$ and $\frac{k}{\sigma_{k}}$. The combined graphs of characteristics of the synchrony of long-term fluctuations in different rivers are presented in Appendix 1.

### 2.3 Andreyanov method

The runoff variations with within year was calculated with Andreyanov method (Andreyanov, 1960). The Andreyanov method was used to estimate the “Surface water resources of the USSR” from 1930 to 1965. The runoff distribution was compared between 1930-1965 and 1965–2019. According to Andreyanov method [[46], [47], [48], [49]], runoff data was considered by months and seasons (as a percentage of annual) for water content periods. Calculation of the distribution of river runoff was carried out for the Lepsy, Sarykan, Koktal, and Byzhy rivers based on Andreyanov method with the variation coefficients of runoff:

(2) $$f\left(t\right)=\frac{\sum_{t=1}^{T}\left(k_{t}-1\right)}{C_{v}}$$

where $C_{v}$ is the variation coefficients of runoff; $k_{t}=\frac{Q_{t}}{Q_{0}}$ is the modulus coefficients; $Q_{t}$ and $Q_{0}$ is the discharge of the $t^{n}$ year and the average discharge for the period of time T. Qualification of water content periods are made according to the probability curves: high-water (25%), medium (50%), low-water (75%), and very low-water (95%).

### 2.4 Mann-Kendall method

The Mann-Kendall test is a statistical method used to detect trends in time series data. The Mann-Kendall test is commonly used in climate and hydrological studies to detect trends in temperature, precipitation, streamflow, and other variables over time [[50], [51], [52], [53]]. The Mann-Kendall test involves calculating a measure of the strength and direction of trends in the data, called the Kendall rank correlation coefficient (tau). If tau is positive, it indicates an increasing trend, while a negative tau indicates a decreasing trend. A tau value of zero indicates no trend. The test additionally calculates a p-value, which indicates the statistical significance of the trend. If the p-value is below a chosen significance level (typically 0.05), it indicates that the trend is significant and unlikely to have occurred by chance. However, it is important to note that the Mann-Kendall test is not a perfect tool and has some limitations. For example, it does not consider the possible influence of other factors on the data, such as changes in land use or natural variability. As with any statistical method, it is important to carefully consider the assumptions and limitations of the Mann-Kendall test and to interpret the results in the context of the specific data and research question at hand.

The Mann-Kendall test was used in this study to assess trends in time series data and find significant or weak changes in climate (temperature and precipitation) and runoff data. The s-statistic is used to test time series with fewer than ten values; for time series with more than ten values, normal approximation or z-statistic are employed. The difference between growing and decreasing pairs of values in the time series is the basis for the s or z-statistic used in the Mann-Kendall test. The formulas for computing Mann-Kendall s-statistic (eg. 3) is:

(3) $$s=\sum_{i=1}^{n-1}\sum_{j=i+1}^{n}sgn\left(x_{j}-x_{i}\right)$$

where $x_{i}$ (i=1,2,…,n−1) and $x_{j}\;\left(j=i+1,\ldots ,n\right)$ are the sequential data values, n is the length of the data series; each of the data point $x_{i}$ is taken as a reference point which is compared with the rest of the data points $x_{j}$ thus, sgn (eg. 4) is expressed as:

(4) $$sgn\;\left(x_{j}-x_{i}\right)=\left\{ \begin{array}{c} +1\;if\;\left(x_{j}-x_{i}\right)>0\\ 0\;if\;\left(x_{j}-x_{i}\right)=0\\ -1\;if\;\left(x_{j}-x_{i}\right)<0 \end{array}\right.$$

The variance statistic (eg. 5) is given as:

(5) $$VAR\;\left(s\right)=\frac{n\left(n-1\right)\left(2n+5\right)-\sum_{i=1}^{m}t_{i}\left(i\right)\left(i-1\right)\left(2i+5\right)}{18}$$

The z - statistic (eg. 6) is computed as:

(6) $$Z=\left\{ \begin{array}{c} \begin{array}{c} \frac{s+1}{\sqrt{var\;\left(s\right)}}\;s>0\\ 0\;s=0 \end{array}\\ \frac{s-1}{\sqrt{var\;\left(s\right)}}\;s\;<0 \end{array}\right.$$

A positive (negative) value of z - statistic indicates an upward (downward) trend. If data is not random and influenced by autocorrelation, modified Mann-Kendall test used for trend detection studies. The Mann-Kendall tests produces false results due to the presence of autocorrelation in time series data; in this case, modified Mann-Kendall tests test gives correct results. The auto correlation coefficient has been calculated by t-test (eg. 7) as:

(7) $$t=\left|P_{1}\right|\sqrt{\frac{n-2}{1-P_{1}^{2}}}$$

where t is the t-value and n is the number of observations. P is value after variance correction which is given by (eg. 8):

(8) $$p=\frac{\sum_{t=1}^{n-k}\left(x_{t}-{\bar{x}}_{t}\right)\left(x_{t+k}-{\bar{x}}_{t+k}\right)}{\sqrt{\left[\sum_{t=1}^{n-k}{\left(x_{t}-{\bar{x}}_{t}\right)}^{2}\sum_{t=1}^{n-k}{(\left(x_{t+k}-{\bar{x}}_{t+k}\right)}^{2})\right]}}$$

where $x_{t}$ is the observed value, ${\bar{x}}_{t}$ is the mean of first *n*-k terms and ${\bar{x}}_{t+k}$ is the mean of last *n*-k terms. If the correlation in the precipitation data has been investigated, the trend analysis has been calculated by modified Mann-Kendall test and a correction factor is given as follow (eg. 9):

(9) $$\frac{n}{n_{S}^{*}}=1+\frac{2}{n\left(n-1\right)\left(n-2\right)}\sum_{k=1}^{n-1}\left(n-k\right)\left(n-k-1\right)\left(n-k-2\right)P_{k})$$

where n is the total observation, $n_{S}^{*}$ is the effective observation for autocorrelation, and $P_{k}$ is the function of automatic correlation. Variance of modified Mann-Kendall test has been calculated by multiplying the variance of Mann-Kendall test by correction factor as (eg. 10):

(10) $$var\left(S*\right)=var\left(S\right)\frac{n}{n_{S}^{*}}$$

Corrected Zc - statistic after variance of modified Mann-Kendall test is computed by using modified variance Var(S*) by following (eg. 11):

(11) $$Zc=\left\{ \begin{array}{c} \frac{s*-1}{\sqrt{var\left(s*\right)}}\;s*>0\\ 0\;s*=0\\ \frac{s*+1}{\sqrt{var\left(s*\right)}}\;s*<0 \end{array}\right.$$

All the statistical analyses were done using open-source software R-studio [54,55].

## 3 Results and analysis

### 3.1 Temperature and precipitation change

**Fig. 2:**
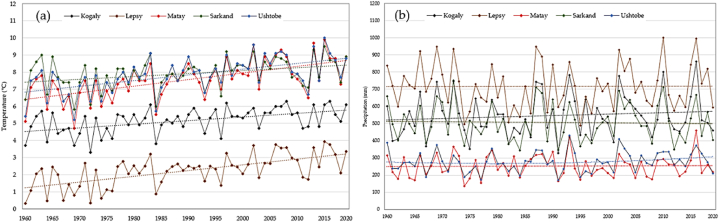
Long-term mean meteorological annual change and its linear trend from 1960 to 2020 in the Zhetysu Alatau, the South-Eastern part of Kazakhstan: (a) temperature, (b) precipitation.

Trends in temperature and precipitation for the period of 1960–2020 are shown in Fig. 2 (a) - (b) and Table 1. The mean air temperature for the period of 1960–2020 varies from 3.3 °C to 6.3 °C in Kogaly station, from 0.3 °C to 3.9 °C in Lepsy, from 4.7 °C to 9.9 °C in the Matay, and 5.8 °C–9.3 °C in Sarkand, and from 5.2 °C to 9.6 °C in Ushtobe. The Mann-Kendall test results show a significant trend (p<0.02) in temperature increases at all stations from 1960 to 2020. The air temperature has increased by a mean of 0.28 °C per decade between 1960 and 2020.

**Table 1:** Results of trend test and change point analyses of temperature and precipitation of the meteorological stations in Kogaly, Lepsy, Matay, Sarkand, Ushtobe.

Meteorological station	Height	Factor	Trend rate	Z-value	Change point^a^	*P*-value
Kogaly	1385 m	Temperature (°C/10 y)	0.22	4.08	1997	0.01
		Precipitation (mm/10 y)	9.34	2.67	1989	0.01
Lepsy	1012 m	Temperature (°C/10 y)	0.27	5.06	1988	0.01
		Precipitation (mm/10 y)	−0.31	−0.31	2003	0.75
Matay	412 m	Temperature (°C/10 y)	0.38	4.74	1990	0.01
		Precipitation (mm/10 y)	1.67	0.07	2003	0.94
Sarkand	948 m	Temperature (°C/10 y)	0.17	2.63	1998	0.01
		Precipitation (mm/10 y)	−2.83	−0.21	2001	0.45
Ushtobe	397 m	Temperature (°C/10 y)	0.34	4.48	1990	0.01
		Precipitation (mm/10 y)	1.68	1.23	2001	0.21

a Change point means a year in a time series where the temperature and precipitation trend have been significantly changed.

In terms of precipitation, at the Kogaly weather station, from 1960 to 2020, there has been an increase in precipitation (p<0.01) by a mean of 9.34 mm per decade, while an insignificant negative trend (p>0.05) is observed at remaining meteorological stations: Lepsy, Matay, Sarkand, Ushtobe. According to the data from weather stations, the mean annual precipitation for the period 1960–2022 was: Kogaly - 546 mm, Lepsy - 718 mm, Matay - 253 mm, Sarkand - 507, Ushtobe - 278 mm.

### 3.2 Temporal runoff dynamics

**Fig. 3:**
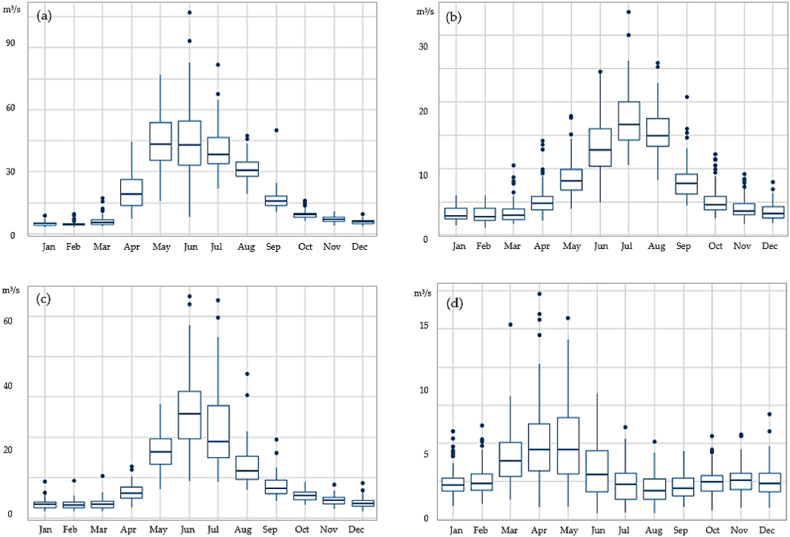
The mean monthly runoff trends for period of 1930–2019 in the Zhetysu Alatau, the South-Eastern part of Kazakhstan for rivers: (a) Lepsy, (b) Sarykan, (c) Koktal, (d) Byzhy.

**Fig. 4:**
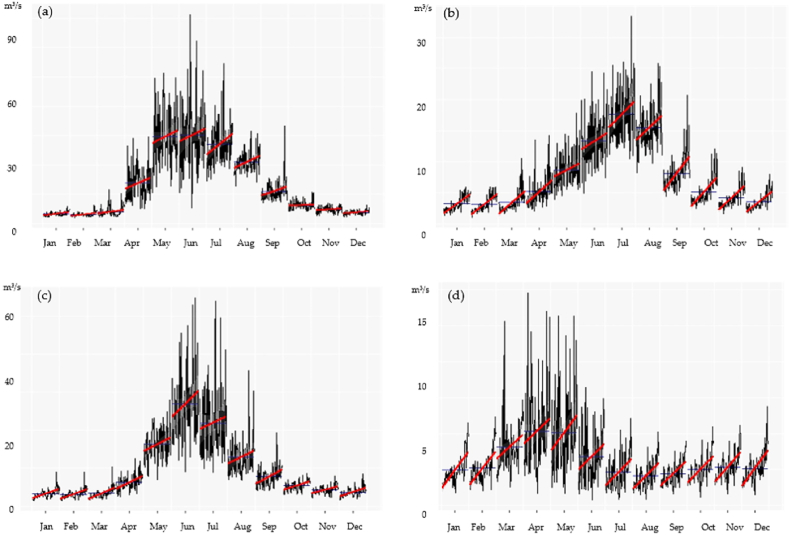
Long-term annual runoff fluctuations for period of 1930–2019 in the Zhetysu Alatau, the South-Eastern part of Kazakhstan for rivers: (a) Lepsy, (b) Sarykan, (c) Koktal, (d) Byzhy.

The change in the mean monthly runoff over a long period of 1930–2019 for Lepsy, Sarykan, Koktal, Byzhy are shown in Fig. 3, and for long-term annual runoff fluctuations are demonstrated in Fig. 3 (a)–(d). The peak of discharge for Lepsy and Sarykan rivers is observed in June and July, for Koktal river in June, and for Byzhy river, measured at the Karymsak station is high in May. The red lines of Fig. 4 (a) - (d) demonstrate a linear regression of increase of runoff for each month. Over a period of 90 years, changes in monthly runoff are clearly visible and the monthly runoff is growing. Seasonal runoff in the study region was tested using a Mann-Kendall test, the results of test are presented in Table 2. A significant positive trend is observed for the Sarykan and Byzhy rivers in all seasons (p<0.02). For Koktal, a significant positive trend season (p<0.05) is observed in the low watery season, from October to February.

**Table 2:** Temporal changes on modified Mann-Kendall test for annual runoff of the different rivers: Lepsy, Koktal, Sarykan, Byzhy.

Hydrological station	March–September			October–November			December–February		
	Zc	*P*-value	Tau	Zc	*P*-value	Tau	Zc	*P*-value	Tau
Lepsy	1.71	0.35	0.08	−1.45	0.28	0.10	0.60	0.44	0.07
Koktal	4.00	0.23	0.11	4.80	0.00	0.29	4.81	0.00	0.37
Sarykan	7.65	0.01	0.23	7.78	0.00	0.54	11.20	0.00	0.57
Byzhy	3.35	0.02	0.22	5.28	0.00	0.37	4.49	0.00	0.49

Note: Zc – Z statistics after variance correction.

### 3.3 Comparative description of change

#### 3.3.1 Lepsy river

**Fig. 5:**
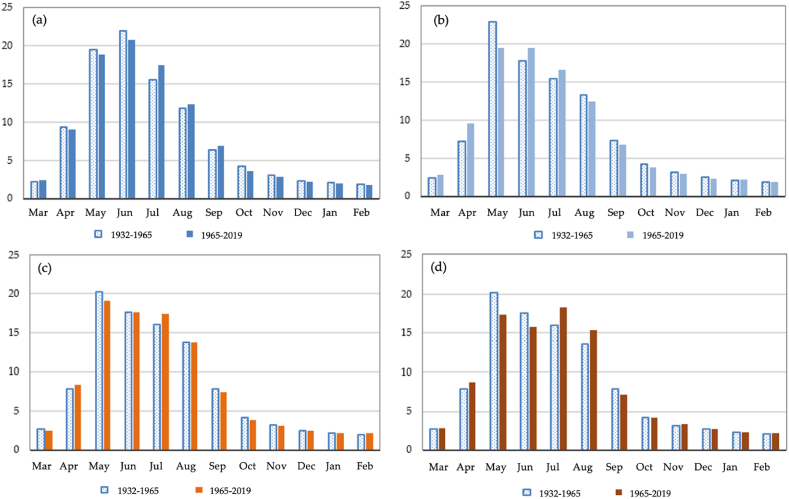
The variability of distribution of observed monthly runoff of the Lepsy river, in % of annual: (a) high watery period, (b) medium watery period, (c) low watery period, (d) very low watery period.

A comparative analysis of the runoff changes for the Lepsy river with a catchment height of 2330 m is presented in Fig. 5 (a) - (d). The Lepsy river of the Zhetysu Alatau is characterized by spring-summer flood runoff. For the period 1932–1965, the distribution of river runoff by hydrologic seasons in a high watery period in terms of the amount of water in the Lepsy river has been evaluated as follows (%, annual volume): spring runoff, 31.0%; summer runoff, 49.2%; fall runoff, 13.5%; winter runoff, 6.3%. For the period 1965–2019, an insignificant decrease in spring, fall, and winter runoff for the monitoring period was 0.8%, 0.2%, and 0.4%, respectively, and an insignificant increase in summer runoff was 1.3%. For the period 1965–2019, there is an insignificant decrease in runoff in May in high watery, low watery, and very low watery periods compared to 1930–1965. On the other hand, there is an increase in runoff in July and August in all four watery periods; insignificant changes are observed in the remaining months. There is a slight increase in runoff in the flood period by 1.1% and a decrease in limit period by 0.8%. Thus, it is concluded that insignificant statistical changes between 1932-1965 and 1965–2019 in intra-annual river runoff are observed.

#### 3.3.2 Sarykan river

**Fig. 6:**
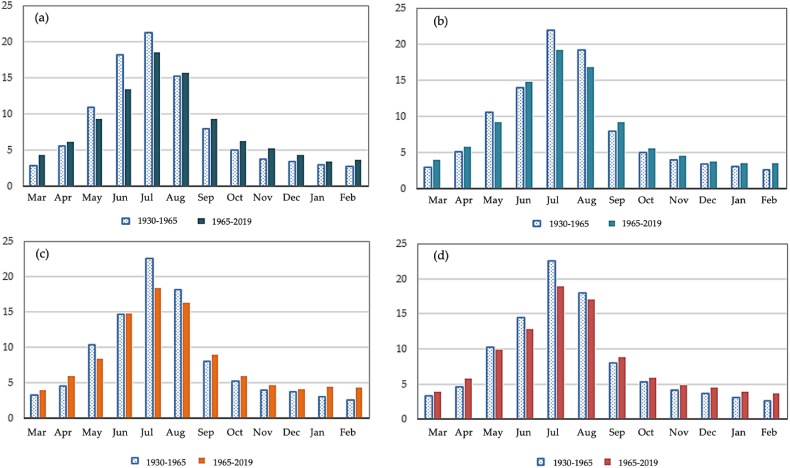
The variability of distribution of observed monthly runoff of the Sarykan river in % of annual: (a) high watery period, (b) medium watery period, (c) low watery period, (d) very low watery period.

A comparative analysis of the runoff change for the Sarykan river with a catchment height of 2490 m is presented in Fig. 6 (a) - (d). Calculations for the period of 1965–2019 show that in the Sarykan river, a decrease in May and summer runoff for the monitoring period was 5.3%, and the increase in runoff remaining months was 6.4% compare to the period of 1930–1965. For the period 1965–2020, a decrease in runoff is observed from May to August in all four watery periods compared to 1930–1965, and from September to April, an increase in runoff is observed. The peak of discharge for all water content groups for 1930–1965 and 1965–2019 is recorded in July. The Sarykan river is characterized by a small share of runoff from December to April and a large share from May to September. There is a decrease in the share of runoff during the flood period (March–September): in high-watery periods by 5% and low-watery and very low-watery periods by 4.8% and 3.8%, respectively. Thus, the Sarykan river has demonstrated statistically significant changes in runoff trends.

#### 3.3.3 Koktal river

**Fig. 7:**
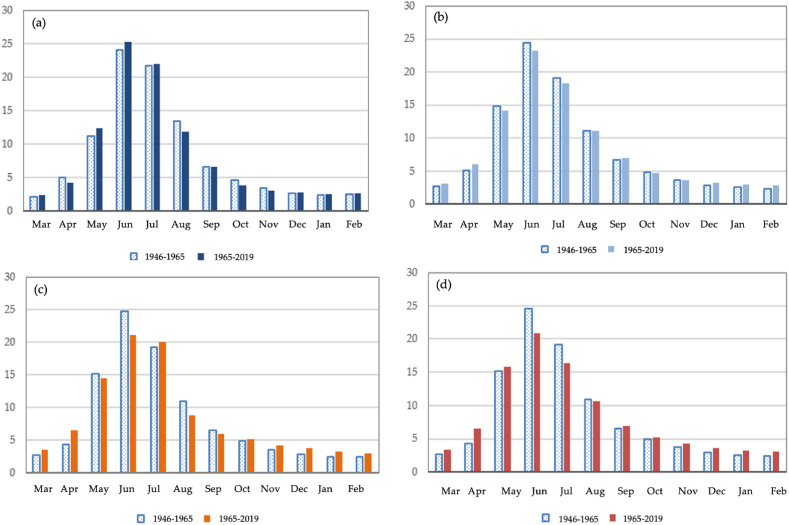
The variability of distribution of observed monthly runoff of the Koktal river, in % of annual: (a) high watery period, (b) medium watery period, (c) low watery period, (d) very low watery period.

The rivers of the northwestern slope of the Zhetysu Alatau are spring-summer flood runoff. The Koktal River (with catchment height of 2910 m) is characterized by low runoff in the autumn-winter month from October to February and high flow in the rest of the year. In a comparative analysis of 1946–1965 and 1965–2019, for the period 1965–2019, there is a slight increase in runoff in May, June, and August in a high watery period, 1.3%, 1.1%, and 1.3% respectively, Fig. 7 (a) - (d). In medium-watery period, there are not significant differences in the distribution of runoff by months. In the low-watery period, an increase in the share of runoff in February–March is noticeable (0.7% and 2.3%), followed by a decrease in runoff in May, June, and July in the low watery period, 0.7%, 3.6%, and 2.8% respectively. In a very low watery period of 1965–2019, the share of runoff in the autumn-winter-spring months slightly increased compared to 1946–1965. The same observations are found for medium and low watery periods. In the summer months (June–July) of the very low watery period, a decrease in the runoff by 3.8% and 2.8% is observed. Generally, there are medium statistically significant changes in runoff in the Koktal river.

#### 3.3.4 Byzhy river

**Fig. 8:**
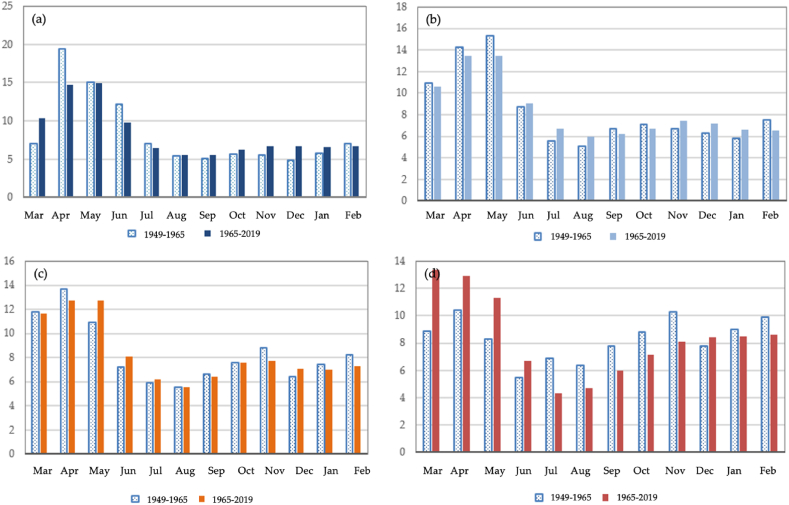
The variability of distribution of observed monthly runoff of the Byzhy river, in % of annual: (a) high watery period, (b) medium watery period, (c) low watery period, (d) very low watery period.

A comparative analysis of the runoff for the Byzhy River with a catchment height of 1490 m is presented in Fig. 8 (a) - (d). From 1949 to 1965, the distribution of river runoff by hydrologic seasons in a high watery period in terms of the amount of water in the Byzhy River has been evaluated as follows (%, annual volume): spring runoff, 41.4%; summer runoff, 25.1%; fall runoff, 16.3%; winter runoff, 17.7%. From 1965 to 2019, an insignificant increase in winter and fall runoff for the monitoring period was 2.2% and 2.0%, and an insignificant decrease in spring and summer runoff was 1.6% and 3.3%. In comparison with the data for 1949–1965 and 1965–2019, in the high-watery period of 1949–1965, a significant decrease in the share of runoff in April by 4.8% is observed. However, in the same low watery period, the share of runoff in the spring months (March–May) and June increased by 4.4, 2.5, and 3.0% more than in 1949–1965. The maximum runoff for all watery periods for 1930–1965 and 1965–2020 is recorded in March, April, and May.

#### 3.3.5 Annual comparison runoff

**Fig. 9:**
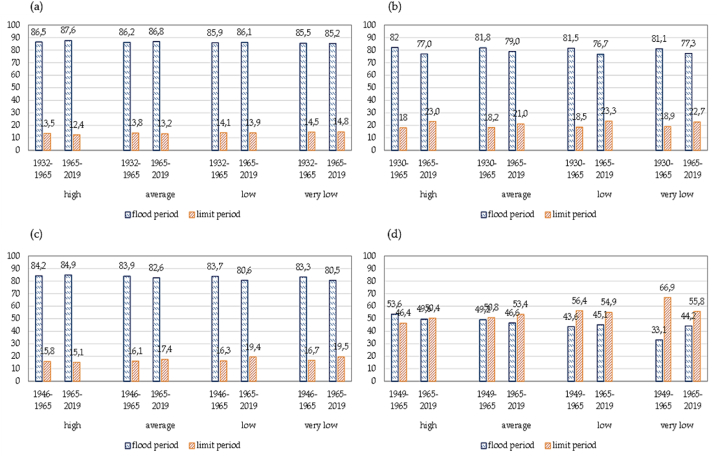
Long-term annual runoff fluctuations in the Zhetysu Alatau, the South-Eastern part of Kazakhstan for rivers, in %: (a) Lepsy, (b) Sarykan, (c) Koktal, (d) Byzhy.

Compared with the high watery periods in 1932–1965 and 1965–2019, there is a slight increase in the share of runoff for Lepsy river by 1.1% (87.6% of the annual runoff) and a decrease in the limiting period by 1.1% (12.4% of the annual runoff). In average and low watery periods, a similar trend is observed. In very low periods, there is a slight increase of 0.3% in the share of runoff in the limiting period, Fig. 9 (a) - (d). The Sarykan river demonstrates fluctuated trends, and for all watery content periods, a decrease in the share of runoff during the flood period is observed. However, in the limiting period, on the contrary, an increase in the runoff by 2.8–5% is noted. In a comparative analysis of the Koktal River for 1965–2019, for all water content periods, except for the high-watery period, there is a decrease in the share of runoff during floods and an increase in limited periods. The share of runoff of the Byzhy River during the flood for 1949–1965 in high watery periods is 53.6% of the annual runoff, and for 1965–2019 it is 49.6% of the annual runoff. There is a decrease in the share of runoff by 4% and an increase in the share of runoff in the limiting period. In average water periods, a similar trend is observed for 1965–2019. There is a decrease in the share of runoff in the flood season (46.6% of the annual runoff) and an increase in the share of runoff in the limiting period by 2.6% (53.5% of the annual runoff).

#### 3.3.6 The correlation analysis

**Fig. 10:**
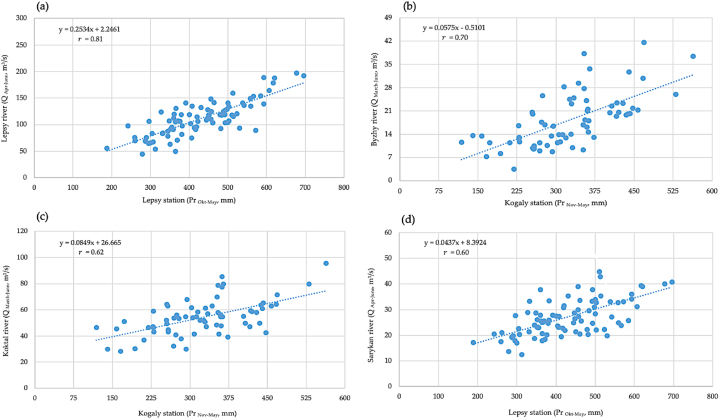
The correlation results between runoff and precipitation: (a) Lepsy river – Lepsy station, (b) Byzhy river – Kogaly station, (c) Koktal river – Kogaly station, (d) Sarykan river - Lepsy station.

In study area of Zhetysu Alatau region, runoff formation separated into spring, summer, autumn and winter periods. It is necessary to pay attention to the role of sources of runoff according to the season of the year. The spring runoff (between April and June) is mainly associated with snowmelt and seasonal precipitations in form of rainfall; summer runoff (between July and September) is linked to seasonal precipitation, the melting of snow, glaciers and ground-ice; autumn runoff (between October and November) is associated with seasonal precipitations in form of rainfall and underground water feeding; winter runoff (between December and March) is associated with underground water feeding and winter thaw events, as well as soil freezing conditions. The precipitation is recorded as one of the main components influencing runoff in hydrological systems of Zhetysu Alatau region from April to December. Fig. 10 (a) - (d) shows the graphs of the relationship between the amount of runoff between April and June for Lepsy river and amount of precipitation for period of October and May from 1993 to 2019 for meteorological station in Lepsy. It is shown that the correlation coefficient is r=0.81, the regression equation is y=0.2534x+2.2461.

The correlation coefficient between runoff in Byzhy river for March and June for the period of 1949 and 2019 and amount of precipitation for November and May for period of 1949 and 2019 in meteorological station in Kogaly is r=0.70, the regression equation has the form y=0.0575x−0.5101. The correlation coefficient between runoff in Koktal river for March–June of period of 1952 and 2019 and amount of precipitation for November–May for period of 1952–2019 in meteorological station in Kogaly, the correlation coefficient is r=0.62, the regression equation is expressed as y=0.0849x+26.665. The correlation coefficient between runoff in Sarykan river for April–June of period of 1933 and 2019 and amount of precipitation for October–May for period of 1932–2019 in meteorological station in Lepsy, the correlation coefficient is r=0.60, the regression equation is expressed as y=0.0437x+8.3924.

## 4 Discussion

Runoff formation, especially in the mountainous and arid territories of Zhetysu Alatau region, depends on many factors. It is important to note that while precipitation is a key factor, other factors such as evaporation, soil moisture, slope of land also influence runoff and overall water availability [56]. In addition, the conditions for river runoff formation in these territories are determined by share of glaciers and the influence of temperature on the mountain cryosphere, including the components of the melting of snow, glaciers, and ground-ice [57]. As it is reported in previous studies, all these cryosphere components experience serios changes under the conditions of the observed temperature trends [58,59]. The glaciers in the studied region have been experiencing significant reduction and retreat in recent decades [60]. Since 1956, the areas of glaciation on the outer and inner ridges the mountain regions of Zhetysu Alatau have decreased from 294.6 km^2^ to 113.1 km^2^ [61], while annual mean surface temperature in region have increased on average 0.29 °C per decade [2]. Furthermore, the runoff of the winter period is largely associated with ground water recharge [18,19]. The widespread steady increase of ground water recharge in the region is often associated with more frequent thaw events, which led to an increase in groundwater reserves [62]. However, a rare network of hydrometeorological monitoring in the study area creates the problems to clearly evaluate the impact of glacier melting on the river runoff. In addition, there is no monitoring on the impact of ground glaciers on river runoff in the region [63]. The absence of monitoring data on the hydrometeorological situation in mountain glacier area have some concerns with making accurate statements [64,65]. Addressing the problems associated with a rare network of hydrometeorological monitoring requires investments in expanding monitoring infrastructure, enhancing data collection and sharing mechanisms, and improving collaboration among relevant agencies and institutions. This helps to improve the accuracy of climate predictions, enhance early warning systems, and enable better-informed decision-making in climate and water resource management.

## 5 Conclusion

This study shows a change in annual and monthly river runoff in the Zhetysu Alatau region, which is highly likely due to the additional inflow of water from the melting zone of the Zhetysu Alatau mountains because of an increase in air temperature than in precipitation. The Sarykan, Byzhy, and Koktal rivers' seasonal runoff have shown a significant positive trend, according to the Mann-Kendall test (p<0.02). The Mann-Kendall test result reveals a statistically significant temperature increase at all meteorological stations (p<0.01), while the fluctuations in precipitation trends are not significant (p>0.05). In connection with the increase in temperature, there was a specific redistribution of runoff each year. The winter thaw became more frequent, and part of the water in the snow formed an additional runoff in the winter months. It reduces the share of runoff at the beginning of the flood, where the role of winter snow reserves is great. The share of runoff during flood decline, when the primary source is snow-glacier resources and permafrost waters, which have large reserves, increased. The percentage of the post-flood era has decreased (the period from the end of the flood to the appearance of a stable snow cover). The characteristic of watery content periods (high, medium, low, and very low watery) and their relationship with current climate change and anthropogenic activity must be studied in more detail for an analysis of the changes that have occurred in the runoff regime in the Zhetysu Alatau region. Additionally, research is required on the long-term dynamics of the primary river sources (liquid and solid precipitation, snow cover, glaciers, permafrost, and groundwater) and their contribution to the development of runoff.

## Author contribution statement

Marat Karatayev; Sabira Issaldayeva; Sayat Alimkulov; Zhanar Raimbekova; Roza Bekseitova: Conceived and designed the experiments; Performed the experiments; Analyzed and interpreted the data; Contributed reagents, materials, analysis tools or data; Wrote the paper.

## Data availability statement

Data will be made available on request.

## Declaration of competing interest

The authors declare that they have no known competing financial interests or personal relationships that could have appeared to influence the work reported in this paper

## Figures and Tables

**Fig. 11:**
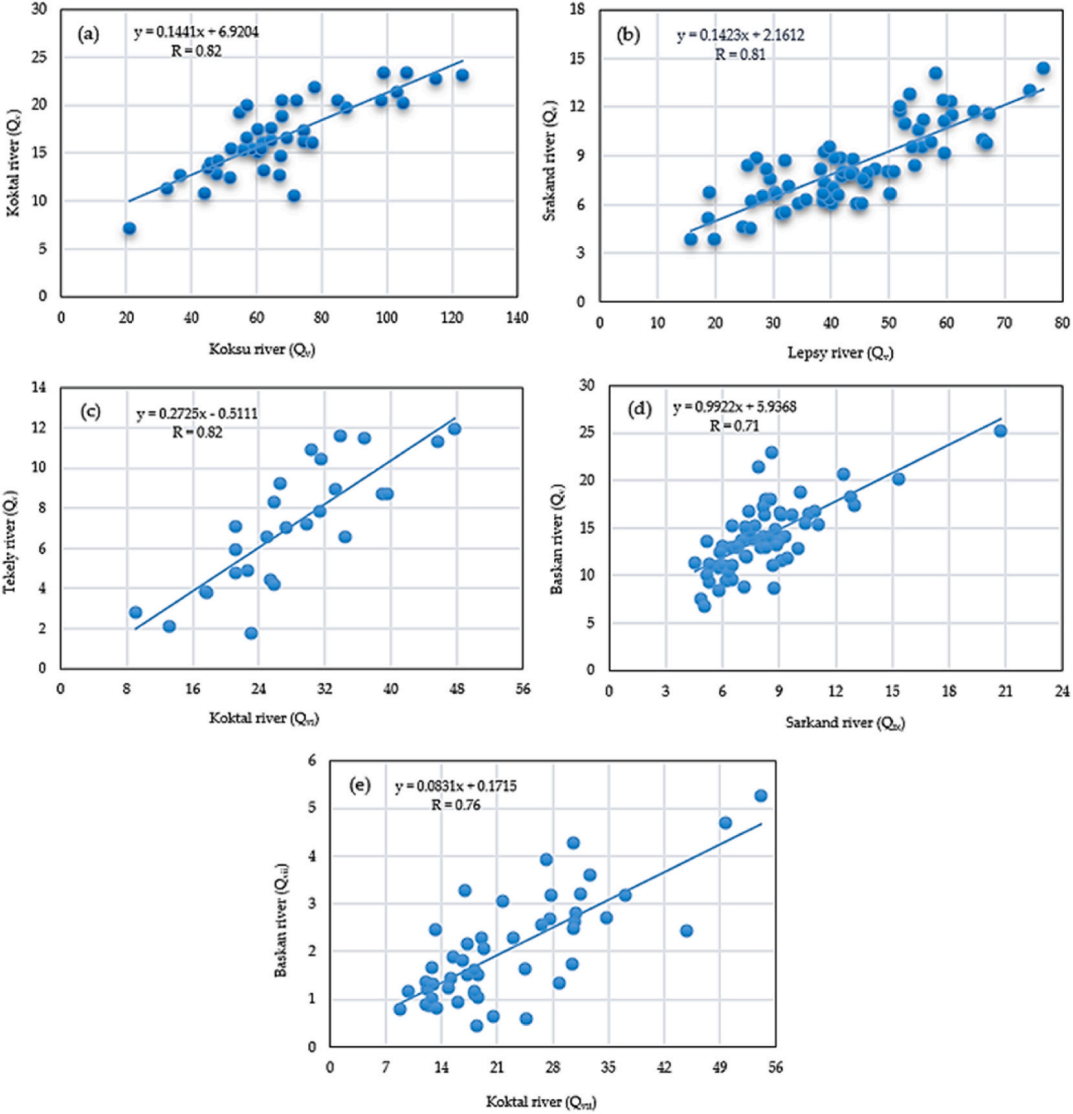
The mean monthly river discharge between reference and analog stations: (a) Koktal - Koksu, (b) Sarkand - Lepsy, (c) Tekely – Koktal, (d) Baskan – Sarkand, (e) Baskan – Koktal.

## Appendix

In hydrology and climatology, analog and reference stations are commonly used for a variety of purposes, including the reconstruction of missing data and comparing data. According to guidelines of SP 33-101-2003 and SNiP 2.01.14–83, the analog stations are those that have similar climatic or hydrological characteristics (e.g., similar catchment area and geographic characteristics, similar condition for runoff formation, slope exposure and hypsometry). When choosing an analog stations, the main criterion is the presence of synchronism in the fluctuations of the river runoff of the calculated site and analog sites, which are quantitatively expressed through the coefficient of pair or multiple correlation between the runoff at these points. Reference stations, on the other hand, are those that are used as benchmarks for comparison or calibration purposes, often because they have long-term records, and other desirable characteristics. In this study, the correlation coefficient between reference and analog station ranges from 0.7 to 0.84, which means that equations reflect a statistically significant relationship between the values of the runoff in reference and analog gauging station. Graphically, it is shown in Fig. 11 (a) - (e). The correlation coefficient for Koktal River (analog point is Koksu River, $Q_{v}$) for May for the period of 1960 and 2003 is r=0.82, the regression equation has the form y=0.144x+6.9204. The correlation coefficient for Sarykan river (analog point is Lepsy river, $Q_{v}$) for May for the period of 1932 and 2001 is r=0.81, the regression equation has the form y=0.1423x+2,1612. The correlation coefficient for the Tekeli River (analog point is Koktal River, $Q_{vi}$) for May for the period of 1970 and 2001 is r=0.82, the regression equation has the form y=0.2725x−0.5111. The correlation coefficient for the Baskan river (analog point is the Sarkand river, $Q_{v}$) for April for the period of 1944 and 2018 is r=0.71, the regression equation has the form y=0.9922x+5.9368. The correlation coefficient for the Baskan river (analog point is Koktal river, $Q_{x}$) for October for the period of 1983 and 2000 is r=0.76, the regression equation has the form y=0.0831x+0.1715. As a control of the validity of the reconstruction of the series, the root-mean-square errors of the reconstructed series of monthly averages were calculated. The value of the root means square error for the reconstructed series is δ = 4.9%, the value of the highest indicator is 9.3% for Tekeli River ($Q_{vii}$), the lowest value (2.8%) is observed for Sarykan River ($Q_{vii}$).

## References

[1] Reyer C.P., Otto I.M., Adams S., Albrecht T., Baarsch F., Cartsburg M., Coumou D., Eden A., Ludi E., Marcus R., Mengel M., Mosello B., Robinson A., Schleussner C.F., Serdeczny O., Stagl J. (2017). Climate change impacts in Central Asia and their implications for development. Reg. Environ. Change.

[2] Karatayev M., Clarke M., Salnikov V., Bekseitova R., Nizamova M. (2022). Monitoring climate change, drought conditions and wheat production in Eurasia: the case study of Kazakhstan. Heliyon.

[3] Malsy M., Aus der, Beek T., Eisner S., Floerke M. (2012). Climate change impacts on Central Asian water resources. Adv. Geosci..

[4] Chen H., Liu H., Chen X., Qiao Y. (2020). Analysis on impacts of hydro-climatic changes and human activities on available water changes in Central Asia. Sci. Total Environ..

[5] Wang X., Chen Y., Li Z., Fang G., Wang F., Liu H. (2020). The impact of climate change and human activities on the Aral Sea Basin over the past 50 years. Atmos. Res..

[6] Deng H., Chen Y. (2017). Influences of recent climate change and human activities on water storage variations in Central Asia. J. Hydrol..

[7] Baspakova G.R., Alimkulov S.K., Sarkynov E.S., Saparova A.A., Kulebayev K.M. (2022). Impact of climate change and anthropogenic factors on the runoff of the Ertis river. Series Geol. Tech. Sci..

[8] Kezer K., Matsuyama H. (2006). Decrease of river runoff in the lake balkhash basin in central Asia. Hydrol. Process.: Int. J..

[9] Huang S., Chen X., Chang C., Liu T., Huang Y., Zan C., Ma X., De Maeyer P., Van de Voorde T. (2022). Impacts of climate change and evapotranspiration on shrinkage of Aral Sea. Sci. Total Environ..

[10] Myrzakhmetov A., Dostay Z., Alimkulov S., Tursunova A., Sarsenova I. (2022).

[11] Bou-Zeid E., El-Fadel M. (2002). Climate change and water resources in the Middle East: a vulnerability and adaptation assessment. ASCE. J. Water Resour. Plann. Manag..

[12] Kriegel D., Mayer C., Hagg W., Vorogushyn S., Duethmann D., Gafurov A., Farinotti D. (2013). Changes in glacierisation, climate and runoff in the second half of the 20th century in the Naryn basin, Central Asia. Global Planet. Change.

[13] Xu C., Chen Y., Chen Y., Zhao R., Ding H. (2013). Responses of surface runoff to climate change and human activities in the arid region of Central Asia: a case study in the Tarim River Basin, China. Environ. Manag..

[14] Lu Y., Xie J., Yang C., Qin Y. (2021). Control of runoff peak flow for urban flooding mitigation. Water.

[15] Tang Q., Lettenmaier D.P. (2012). 21st century runoff sensitivities of major global river basins. Geophys. Res. Lett..

[16] Pieczonka T., Bolch T. (2015). Region-wide glacier mass budgets and area changes for the Central Tien Shan betweeñ 1975 and 1999 using Hexagon KH-9 imagery. Global Planet. Change.

[17] Huss M., Hock R. (2018). Global-scale hydrological response to future glacier mass loss. Nat. Clim. Change.

[18] Sorg A., Bolch T., Stoffel M., Solomina O., Beniston M. (2012). Climate change impacts on glaciers and runoff in Tien Shan (Central Asia). Nat. Clim. Change.

[19] Sorg A., Mosello B., Shalpykova G., Allan A., Clarvis M.H., Stoffel M. (2014). Coping with changing water resources: the case of the Syr Darya river basin in Central Asia. Environ. Sci. Pol..

[20] Chigrinets A., Duskayev K., Mazur L., Chigrinets L., Akhmetova S., Mussina A. (2020). Evaluation and dynamics of the glacial runoff of the rivers of the Ile Alatau northern slope in the context of global warming. Int. J. Eng. Res..

[21] Alimkulov S., Tursunova A., Kulebaev K., Zagidullina A., Myrzahmetov A., Saparova A. (2019). Resources of river runoff of Kazakhstan. Int. J. Eng. Adv. Technol..

[22] Olsson O., Gassmann M., Wegerich K., Bauer M. (2010). Identification of the effective water availability from streamflows in the Zerafshan river basin, Central Asia. J. Hydrol..

[23] Imentai A., Thevs N., Schmidt S., Nurtazin S., Salmurzauli R. (2015). Vegetation, fauna, and biodiversity of the Ile delta and southern lake balkhash—a review. J. Great Lake. Res..

[24] Medeu A. (2015). The methodology of natural hazards management in Kazakhstan. Geogr. Environ. Sustain..

[25] Bernauer T., Siegfried T. (2012). Climate change and international water conflict in Central Asia. J. Peace Res..

[26] Tursunova A., Medeu A., Alimkulov S., Saparova A., Baspakova G. (2022). Water resources of Kazakhstan in conditions of uncertainty. J. Water Land Dev..

[27] Pomfret R., Anderson K. (2001). Economic development strategies in central Asia since 1991. Asian Stud. Rev..

[28] Karatayev M., Kapsalyamova Z., Spankulova L., Skakova A., Movkebayeva G., Kongyrbay A. (2017). Priorities and challenges for a sustainable management of water resources in Kazakhstan. Sustain. Water Qual. Ecol..

[29] Rivotti P., Karatayev M., Mourão Z.S., Shah N., Clarke M.L., Konadu D.D. (2019). Impact of future energy policy on water resources in Kazakhstan. Energy Strategy Rev..

[30] Karthe D., Chalov S., Borchardt D. (2015). Water resources and their management in central Asia in the early twenty first century: status, challenges and future prospects. Environ. Earth Sci..

[31] Duskayev K., Myrzakhmetov A., Zhanabayeva Z., Klein I. (2020). Features of the sediment runoff regime downstream the Ile river. J. Ecol. Eng..

[32] Abdrahimov R., Amirgaliyeva A., Tastambek K., Zhumalipov A., Polyakova S. (2020). Annual river runoff of the Ile-Balkash basin and prospects of its assessment due to climatic changes and water economy activities. GEOMATE J..

[33] NAK (2022).

[34] Vilesov E.N., Naumenko A.A., Veselova L.K., Aubekerov B.Zh (2009). UDK.

[35] Kaldybayev A., Chen Y., Issanova G., Wang H., Mahmudova L. (2016). Runoff response to the glacier shrinkage in the Karatal river basin, Kazakhstan. Arabian J. Geosci..

[36] Kaldybayev A., Chen Y., Vilesov E. (2016). Glacier change in the karatal river basin, Zhetysu (dzhungar) Alatau, Kazakhstan. Ann. Glaciol..

[37] Vilesov E.N., Seversky I.V. (2015). Degradation of glaciers in Dzungarian (Zhetisu) Alatau in the second half of the XX century. Ice and Snow.

[38] Valeyev A., Karatayev M., Abitbayeva A., Uxukbayeva S., Bektursynova A., Sharapkhanova Z. (2019). Monitoring coastline dynamics of Alakol Lake in Kazakhstan using remote sensing data. Geosciences.

[39] Sala R., Deom J.M., Aladin N.V., Plotnikov I.S., Nurtazin S. (2020). Large Asian Lakes in a Changing World.

[40] Duan W., Zou S., Chen Y., Nover D., Fang G., Wang Y. (2020). Sustainable water management for cross-border resources: the Balkhash Lake basin of central Asia, 1931–2015. J. Clean. Prod..

[41] Shek N.D. (1970). Central and Southern Kazakhstan.

[42] Zygas M., Jezierski G. (1999). Probable mean monthly, half-yearly, and yearly discharges in the Tywa River basin (1961-1995). Baltic Coastal Zone. J. Ecol. Protect. Coastline.

[43] Kuhn M. (1993).

[44] Wang W., Hu S., Li Y., Cao S. (2013). How to select a reference basin in the ungauged regions. J. Hydrol. Eng..

[45] Davletgaliev S.K., Alimkulov S.K., Talipova E.K. (2020). The possibility to applying simulated series for compile scenario forecasting river runoff. Environ. Earth Sci..

[46] Andreyanov V.G., Андреянов В.Г. (1960). Внутригодовое распределение речного стока.

[47] Dmitrieva V.A. (2011). Intraannual and multiyear dynamics of seasonal river runoff. Arid Ecosyst..

[48] Fedorova I., Chetverova A., Bolshiyanov D., Makarov A., Boike J., Heim B., Morgenstern A., Overduin P., Wegner C., Kashina V., Eulenburg A., Dobrotina E., Sidorina I. (2015). Lena Delta hydrology and geochemistry: long-term hydrological data and recent field observations. Biogeosciences.

[49] Frolova N.L., Agafonova S.A., Kireeva M.B., Povalishnikova E.S., Pakhomova O.M. (2017). Recent changes of annual flow distribution of the Volga basin rivers. Geogr. Environ. Sustain..

[50] Bae D.H., Jung I.W., Chang H. (2008). Long‐term trend of precipitation and runoff in Korean river basins. Hydrol. Process.: Int. J..

[51] Fathian F., Dehghan Z., Bazrkar M.H., Eslamian S. (2016). Trends in hydrological and climatic variables affected by four variations of the Mann-Kendall approach in Urmia Lake basin, Iran. Hydrol. Sci. J..

[52] Güçlü Y.S. (2018). Multiple Şen-innovative trend analyses and partial Mann-Kendall test. J. Hydrol..

[53] Nyikadzino B., Chitakira M., Muchuru S. (2020). Rainfall and runoff trend analysis in the Limpopo River basin using the Mann Kendall statistic. Phys. Chem. Earth, Parts A/B/C.

[54] Markova R., Xhaja A. (2016). The analysis of the seasonality and trends of the annual maximum discharges in the upper Danube River. Int. Multidiscipl. Sci. GeoConf.: SGEM.

[55] Vardanyan T.G., Frolova N.L., Galstyan H.S. (2021). The characteristics of extreme maximum runoff of the rivers of Armenia in the context of global climate change. Geogr. Environ. Sustain..

[56] Grigorev V.Y., Kharlamov M.A., Semenova N.K., Sazonov A.A., Chalov S.R. (2023). Impact of precipitation and evaporation change on flood runoff over Lake Baikal catchment. Environ. Earth Sci..

[57] Vilesov E.N., Seversky I.V., Morozova V.I. (2015). Dynamics of glaciation in the Kazakh part of Altai during 60 years. Ice and Snow.

[58] Aizen V.B., Kuzmichenok V.A., Surazakov A.B., Aizen E.M. (2006). Glacier changes in the central and northern Tien Shan during the last 140 years based on surface and remote-sensing data. Ann. Glaciol..

[59] Bolch T. (2007). Climate change and glacier retreat in northern Tien Shan (Kazakhstan/Kyrgyzstan) using remote sensing data. Global Planet. Change.

[60] Seversky I.V., Shesterova I.N., Северский И.В., Шестерова И.Н. (2011). Влияние деградации горного оледенения на гидрологический режим и водные ресурсы. Вопросы географии и геоэкологии.

[61] Seversky I.V., Vilesov E.N., Kokarev A.L., Shesterova I.N., Morozova V.I., Kogutenko L.V., Usmanova Z.S., Северский И. В., Вилесов Е.Н., Кокарев А.Л., Шестерова И.Н., Морозова В.И., Когутенко Л.В., Усманова З.С. (2012). Ледниковые системы Балкаш-Алакольского бассейна: состояние, современные изменения.

[62] Mohammed A.A., Pavlovskii I., Cey E.E., Hayashi M. (2019). Effects of preferential flow on snowmelt partitioning and groundwater recharge in frozen soils. Hydrol. Earth Syst. Sci..

[63] Gershtein G.G., Schaffhauser A., Kienberger S. (2022). http://worldbank.org.

[64] Severskiy I., Vilesov E., Armstrong R., Kokarev A., Kogutenko L., Usmanova Z., Morozova V., Raup B. (2016). Changes in glaciation of the Balkhash–Alakol basin, central Asia, over recent decades. Ann. Glaciol..

[65] Klein I., Dietz A.J., Gessner U., Galayeva A., Myrzakhmetov A., Kuenzer C. (2014). Evaluation of seasonal water body extents in Central Asia over the past 27 years derived from medium-resolution remote sensing data. Int. J. Appl. Earth Obs. Geoinf..

